# Heat-processed rodent chow alters nutritive content and improves metabolic outcomes in insulin-resistant female mice

**DOI:** 10.3389/fnut.2026.1829397

**Published:** 2026-06-08

**Authors:** Adelaide E. Weidner, Kenji Vann, Denise Ivey, Zachary R. Sechrist, Calvin L. Cole, Olga Astapova

**Affiliations:** 1 Department of Medicine, Division of Endocrinology, University of Rochester Medical Center, Rochester, NY, United States; 2 Department of Environmental Medicine and Public Health Sciences, University of Rochester Medical Center, Rochester, NY, United States; 3 Department of Pathology and Laboratory Medicine, University of Rochester Medical Center, Rochester, NY, United States; 4 Department of Surgical Oncology, University of Rochester Medical Center, Rochester, NY, United States; 5 Department of Orthopedics, Center for Musculoskeletal Research, University of Rochester Medical Center, Rochester, NY, United States

**Keywords:** animal models, autoclave, glucose, insulin resistance, metabolism, rodent chow, sugar, ultra-processed foods

## Abstract

**Introduction:**

Animal research diets are often autoclaved for sterilization. Autoclaved chow can also serve as a model for investigating the health effects of heat-processed foods. Emerging evidence finds that heat-processed foods adversely affect human health. We hypothesized that autoclaved chow would further impair metabolism in a mouse model of androgen-induced insulin resistance.

**Methods:**

C57BL/6J mouse breeding pairs were exposed to an autoclaved chow diet or a non-autoclaved, nutrient-matched control chow starting 2 weeks before mating and continuing through mating, pregnancy, and lactation. Diets were characterized by macronutrient and B vitamin analysis (B1, B5, B6), methylglyoxal and advanced glycation end products (ELISA), and reducing sugar content. Female 21-day-old weanlings received a 90-day continuous-release subcutaneous dihydrotestosterone implant to induce a polycystic ovary syndrome (PCOS)-like phenotype characterized by diet-independent, non-obese insulin resistance. Mice were kept on the assigned diet until experimentation 90 days post-androgenization. Metabolism was evaluated by measuring adiposity, fasting blood glucose and insulin, and glucose and insulin tolerance. Tissue markers of insulin signaling, methylglyoxal detoxification (GLO1), and inflammation (IL-6) were assayed via Western blot or quantitative PCR.

**Results:**

Consistent with previous literature, PCOS mice on the non-autoclaved diet developed excess adiposity, glucose intolerance, and insulin resistance. Unexpectedly, the autoclaved chow improved adiposity, glucose tolerance, and fasting insulin in PCOS mice. Diet characterization revealed no differences in macronutrients and no B vitamin deficiency in either diet, but a significant decrease in sugar content and a corresponding increase in methylglyoxal due to autoclaving. The autoclaved diet differentially modulated insulin signaling markers in skeletal muscle and adipose and GLO1 expression in liver. IL-6 expression was unaffected by diet in liver and adipose.

**Discussion:**

Autoclaved rodent chow improved the metabolic outcomes of a mouse model of PCOS. Despite nutrient-matching of the autoclaved diet by the manufacturer, we identified autoclave-induced discrepancies in sugar content and methylglyoxal. The autoclaved diet had tissue-specific effects on molecular markers of insulin signaling and methylglyoxal detoxification in PCOS mice, suggesting that diet preparation may be confounding animal studies. Our findings suggest that researchers should thoroughly validate and report dietary parameters in animal studies and consider them when interpreting and replicating findings.

## 1 Introduction

Heat processing is a ubiquitous method for the preparation, preservation and sterilization of foods, commonly used to increase food safety and extend its shelf life. Research animal diets are often heat-processed by autoclaving, either to pasteurize diets and control microbial contamination or as animal models of ultra-processed or high-advanced glycation end product (AGE) diets (1, 2). It is increasingly clear that these practices may introduce variation into animal studies by inducing undesired and likely yet-uncharacterized effects in the chow that have biological implications (3, 4), thus confounding results and impeding replicability. Further, diet sterilization methods are frequently altered, whether intentionally as an experimental model, for animal husbandry reasons at the institution level, or by the diet manufacturers without researchers' knowledge, severely limiting the scientific rigor of such studies' findings (5).

Heat processing alters the chemical composition of some nutrients through degradation or chemical modifications including oxidation and glycation (6–10) and induces the formation of toxic contaminants, such as acrylamide (11–13) and AGEs (14) and their reactive precursors, α-dicarbonyl compounds (e.g. methylglyoxal). This can alter the digestibility of certain starches, decrease nutrient bioavailability (15–17), and trigger adverse tissue-level responses such as inflammation (18, 19) and tumorigenesis (20, 21), negatively affecting health parameters (22). Acrylamide, for example, has carcinogenic properties and is linked to cancers of the lung, breast and prostate (23).

Heat-processed foods have been linked to obesity, insulin resistance, and polycystic ovary syndrome (PCOS) both in observational and randomized, controlled studies (6, 24–31). PCOS is a common endocrinopathy that affects approximately 10% of women and is characterized by hyperandrogenism, anovulation and systemic insulin resistance (32, 33). While the pathogenesis of PCOS is multifactorial and partly genetic (34–36), diet significantly modulates PCOS risk and its manifestations (37), and growing evidence links heat-processed food to PCOS through the accumulation of AGEs (38–41). Of note, a clinical trial found significant improvement in glucose metabolism in overweight women after 4 weeks of a low-AGE diet, even after adjusting for macronutrient intake, age, and change in weight (42).

AGEs are stable, covalently modified proteins formed as a result of the Maillard reaction between reducing sugars, amino acids, and heat. Methylglyoxal (MG) is a reactive α-dicarbonyl precursor of AGEs which can be produced by heat degradation of glucose or endogenously during glycolysis. In healthy organisms, MG is detoxified by the antioxidant enzymes glyoxalase 1 (GLO1) and glyoxalase 2 (GLO2). However, α-dicarbonyls, AGEs, and glyoxalases are modulated by diet and metabolic state, and the capacity of this detoxifying system can be overwhelmed by excessive α-dicarbonyl exposure or in chronic disease conditions (43), leading to AGE-mediated inflammation and tissue damage.

The aim of this study was to determine the effects of a heat-processed research diet on the metabolic outcomes in a mouse model of PCOS. To determine the effects of heat processing without nutrition as a confounder, we developed a set of nutrient-matched experimental rodent diets: one of which was heat-processed via autoclaving (“autoclaved chow”); the other an unheated, nutrient-matched control diet (“non-autoclaved chow”). To account for micronutrient degradation due to autoclave heating, the autoclaved diet was supplemented by the manufacturer with higher levels of heat-sensitive vitamins. We applied these experimental diets to a mouse model of PCOS induced by chronic androgen exposure. We and others have found that this mouse model develops diet-independent, non-obese insulin resistance (44, 45). Female offspring were exposed to these experimental diets beginning 2 weeks before conception and throughout gestation, lactation, and adulthood in a broad developmental exposure paradigm. We hypothesized that prolonged exposure to heat-processed chow would cause worsened metabolic outcomes in the insulin-resistant PCOS mice. Unexpectedly, the autoclaved chow exerted a consistent improvement in the glucose metabolism of PCOS mice compared to the nutrient-matched, non-autoclaved chow. We then characterized the nutritive content of the chow and found that autoclaving altered the sugar and methylglyoxal composition in the diet, modulating the PCOS effect on tissue markers of insulin signaling. Our results indicate that a heat-processed diet improves metabolic outcomes in this insulin-resistant mouse PCOS model and that reporting of nutritional content in research diets may be inaccurate, contributing to experimental pitfalls such as impaired reproducibility and unexplained findings.

## 2 Materials and methods

### 2.1 Animals

Animal experimentation in the present study adheres to the ARRIVE 2.0 guidelines for animal research reporting (46). C57BL/6J mice were obtained from Jackson Laboratory, and the colony was bred and maintained in the AAALAC International-accredited Vivarium facilities at the University of Rochester Medical Center (URMC). Animals were housed in ventilated cage racks (Allentown JAG system) in husbandry rooms with a 12-h light/dark cycle with lights on at 6:00 AM. Rodent husbandry rooms were maintained at 74°F and 30–70% relative humidity according to the ILAR Guide for the Care and Use of Laboratory Animals (1996). Husbandry services (cage changes, chow preparation) for animals in this study were provided by the investigators. Animals were provided *ad libitum* food and water at all times, except while fasting prior to metabolic experimentation and sacrifice. Ultra-filtered (0.1 μm) Rochester, NY municipal water was provided in Hydropac pouches (Avidity Science). Cages were changed weekly and were each supplied with 1/8” pelleted cellulose bedding (Biofresh Performance Bedding), 2 cotton nestlet squares (Ancare #NES3600), and a plastic house (Bio-Serv #K3583). Animal wellness was monitored at least every 3 days by the URMC Vivarium staff. All experiments involving research animals were approved by University Committee on Animal Resources at the University of Rochester.

### 2.2 Experimental diets & autoclave sterilization

Rodent chow diets were purchased from Inotiv (Madison, WI) and stored in airtight containers at 4 °C upon receipt. Non-autoclaved chow (Teklad #2018) was provided to animals without manipulation. Autoclaved chow (Teklad #2018S) was prepared by autoclaving in an uncovered aluminum tray lined with aluminum foil (Fisherbrand #15–078–292) using a Vacuum Steam Sterilizer (Getinge, model #633LS). The autoclave cycle consisted of a 30-min gravity steam exposure at 121 °C / 30 PSIA followed by a 30-min gravity drying stage at 110 °C / 14 PSIA. Autoclaved chow was cooled to room temperature overnight before feeding to animals. Chow was autoclaved fresh each week on the day before weekly cage changes. Experimenters were blinded to mouse diet assignments when possible, i.e. during fasted metabolic experiments, but blinding was not always possible due to the difference in appearance of the autoclaved and non-autoclaved chow present in mouse cages.

### 2.3 Dietary exposure

Proven-fertile dams and sires between 13 and 20 weeks of age were bred to generate offspring for the described experiments. Prior to starting the experimental diets, all breeders were fed standard Vivarium chow (LabDiet #5053, PicoLab Rodent Diet 20). Dams and sires were randomly assigned to the autoclaved or non-autoclaved diet, acclimated to the diet for 2 weeks, then paired for 1 week to mate, after which the male was removed and the female monitored for pregnancy and parturition. Mice remained on the assigned experimental diet throughout gestation and lactation, and female offspring were weaned onto the same diet on P21. To provide nutritional support on the day of weaning, pups were provided one Nutragel cup per cage (Bio-Serv #NGB-2). This 1–2 day duration of transitional food is not expected to significantly affect the study results. Due to the choice of experimental model of PCOS, male offspring were excluded from this study.

### 2.4 PCOS mouse model

As previously described (44), female P21 mice were anesthetized using 1–5% vaporized veterinary-grade isoflurane. Aseptic surgical technique was used to insert a 2.5 mg dihydrotestosterone (DHT) slow-release pellet or placebo pellet (Innovative Research of America #NA-161 and #C-111) into the subcutaneous space dorsally at the shoulder blades. The small incision was secured with a single suture, and animals were monitored closely during recovery. Female offspring within each litter were randomly assigned to DHT or placebo treatment to account for potential litter-specific effects. Following weaning and pellet insertion, animals were group-housed by treatment in cages of 2–3 mice, except during estrous cycling experiments. Experimentation was performed at 15–16 weeks of age (90 days after androgenization) unless otherwise specified. Blinding of experimenters to DHT treatment was not possible due to differences in appearance and behavior of DHT-treated mice.

### 2.5 Food consumption and body weight measurements

The investigators provided mice with a clean cage and fresh chow and water once every 7 days throughout the duration of the study. During weekly cage changes, body weight of each mouse was recorded, and chow was weighed to calculate food intake per cage. The investigators did not observe an appreciable degree of chow shredding with either of the experimental diets.

### 2.6 Body composition

Body fat and lean mass percentages were measured as previously described (45). Briefly, mice were imaged under anesthesia at 14–15 weeks of age in a Dual Energy X-ray Absorptiometry (DEXA) scanner (iNSiGHT VET DXA, OsteoSys) after a 2-h food and water fast.

### 2.7 Estrous cycling

Mouse estrous cycles were assessed via cytology of vaginal lavage samples, which were collected from 12–14-week-old mice daily before 12:00 PM for 14 consecutive days, as well as prior to sacrifice to verify diestrus. Mice were single-housed 1 week prior to and throughout the 14-day cycling period. Estrous stages were determined by cytology of lavage samples as previously described (47, 48).

### 2.8 Ovarian morphology analysis

One ovary from each mouse was dissected at sacrifice and fixed overnight in 10% neutral buffered formalin, which was replaced the following day with 70% ethanol. Whole ovaries were paraffin-processed and 5-μm sections were mounted on slides by the University of Rochester Medical Center Histology Laboratory. Slides were baked at 60 °C for 20 min before deparaffinization and rehydration followed by staining in hematoxylin (1 min, Epredia #6765009) and eosin (5 min, Epredia #6766007). Slides were then dehydrated and coverslips mounted with Cytoseal XYL (Epredia #8312–4). One section per mouse was imaged with a bright-field microscope. Theca cell layer thickness of all medium and large antral follicles per section was measured using ImageJ (NIH, Version 1.53k). Two separate measurements per follicle, separated by 90°, were recorded. All follicle measurements per mouse were averaged and reported as a single data point for each mouse.

### 2.9 Granulosa cell estradiol synthesis

Primary granulosa cells (GCs) from mouse ovaries were isolated at sacrifice and cultured as previously described (45). Cells were serum-starved 2 days after plating and treated the following day with testosterone (50 nM, Steraloids #A6950–000) with or without follicle-stimulating hormone (FSH, 100 ng/ml, Sigma-Aldrich #869001-M) for 40 h before media was collected for analysis. GC estradiol synthesis was measured in media samples via ELISA (CalBioTech #ES380S, lot #ESS0100, RRID: AB_3740017).

### 2.10 Glucose tolerance testing (GTT)

A 20% w/v solution of glucose (dextrose) in water was sterilized with a 0.2 μm syringe filter. At 14–15 weeks of age, animals were injected intraperitoneally with 2 g glucose per kg body weight (200–300 μl injection volume) following a 6-h fast. Blood for glucose measurements was sampled from the tail vein at 0, 15, 30, 60, and 120 min following glucose injection using a veterinary glucometer on the dog setting (AlphaTrak3, Zoetis). One control mouse on the non-autoclaved diet, one PCOS mouse on the non-autoclaved diet, and one PCOS mouse on the autoclaved diet were excluded from GTT analysis due to an absent blood glucose spike following injection (defined as < 50% maximum increase from fasting blood glucose).

### 2.11 Insulin tolerance testing (ITT)

A 0.05 U/ml solution of insulin (Humulin R U-100, Lilly) was prepared in sterile phosphate-buffered saline (PBS). At 14–15 weeks of age, animals were injected intraperitoneally with 0.5 U insulin/kg body weight (200–300 μl injection volume) following a 6-h fast. Blood glucose was measured as described above for GTT. Mice were monitored for symptoms of hypoglycemia (blood glucose < 55 mg/dl) to be removed from experimentation and injected intraperitoneally with 2 g/kg glucose solution; however, no animals reached this threshold.

### 2.12 Mouse tissue and serum collection

Estrous cycles were determined as described above, and mice were euthanized during diestrus at 16 weeks of age by CO_2_ inhalation followed by cervical dislocation at 2:00 PM after a 6-h fast. Whole blood was collected via cardiac puncture and allowed to clot at room temperature for 15–30 min. Serum was separated by centrifuging clotted blood at 2,000 x g for 10 min at 4 °C, then stored at −20 °C. 5–10 mg whole tissue samples for protein and RNA isolation were dissected at sacrifice and immediately flash-frozen in liquid nitrogen before storing at −80 °C until processing and analysis.

### 2.13 Fasting glucose and insulin measurements

Fasting insulin was measured via mouse insulin ELISA (Crystal Chem #90080, low-range assay, lot #23DEUMI744, RRID: AB_2783626) in 6-h fasted serum collected at sacrifice as described above. Fasting blood glucose measurements were obtained during GTT (0 min readings). HOMA-IR was calculated for each mouse by dividing the product of fasting glucose (mg/dl) and fasting insulin (mU/L) by 405. Fasting insulin was converted to mU/L by dividing the value in pmol/L by 6 and assuming a molecular weight of 5808 g/mol for insulin.

### 2.14 Chow vitamin and macronutrient measurements

Chow samples were analyzed for vitamin B1 (thiamine), B5 (pantothenic acid), B6 (pyridoxine), and macronutrient content by an independent food testing laboratory, EMSL Analytical (Cinnaminson, NJ). Proximates analysis provided measurements for energy density, total carbohydrates, fat, and protein. Fat was obtained via Soxhlet extraction. B vitamins were quantified by LCMS/MS (LOQ = 0.002–0.010 mg/100 g). Manufacturer-reported levels of total carbohydrates were calculated as the sum of available carbohydrates and neutral detergent fiber (49, 50).

### 2.15 Chow MG and AGE measurements

Mouse food extracts were prepared as previously validated (20): chow samples were freeze-dried (Labconco Freezone Triad Freeze Dry System, model #7400040) and stored at −80 °C until processing. Individual freeze-dried chow pellets were ground to a powder in a mortar and pestle and analyzed separately as replicates. 250 mg powder from each chow pellet was vortexed in 2 ml PBS for 48 h at 4 °C. Chow extracts were clarified twice by centrifugation at 13,000 x g for 20 min at 4 °C and filtered through 0.45 μm syringe filters before storing at −20 °C until analysis. Methylglyoxal (MG) and advanced glycation end products (AGEs) were measured in chow extracts via ELISA (MG: Abcam #ab238543, lot #1132944–1, RRID: AB_3740015; AGE: Abcam #ab238539, lot #1060968–1, RRID: AB_3740016).

### 2.16 Chow reducing sugar measurements

A quantitative adaptation of Benedict's test (51) was followed to determine reducing sugar content in chow extracts prepared as described above. A standard curve of glucose (0, 0.25, 2.5, 5, and 10 mg/ml) was used to relatively quantify unknown samples. 250 μl sample or standard was assayed per 1 ml Benedict's reagent.

### 2.17 Western blot

A 5–10 mg whole tissue samples (liver, skeletal muscle (right quadriceps), and white adipose (perirenal)) were collected at sacrifice as described above and homogenized in 500 μl of RIPA buffer (Thermo Scientific #89900) plus protease/phosphatase inhibitor (Thermo Scientific #78440) using a pellet pestle (DWK Life Sciences #749540) in 1.5-ml microcentrifuge tubes, with the exception of adipose, which was homogenized in 400 μl buffer. Homogenized samples were clarified by centrifugation at 15,000 x g for 10 min at 4 °C and stored at −20 °C. Protein content for Western blot was determined by BCA (Thermo Scientific #23227) and used to calculate sample loading volumes for 10–15 μg protein per lane. An equal amount of protein was loaded in all lanes for each tissue. Western blot was performed as described previously (45). Membranes were incubated in primary antibody overnight at 4 °C. Primary antibodies were diluted in blocking buffer (5% w/v dry nonfat milk/TBST) according to concentrations in Table 1. Blots were incubated in secondary antibody (Bio-Rad #1706515) diluted 1:5,000 in blocking buffer for 1 h at room temperature and imaged with ECL substrate (Bio-Rad #1705061 or #1705062) in a ChemiDoc XRS+ imaging system (Bio-Rad). Band density was quantified using ImageJ (NIH, Version 1.53k). Expression of INSR and GLO1 was normalized to GAPDH; phosphorylated AKT (pAKT) was normalized to total AKT expression.

**Table 1:** Primary antibody product information, concentrations, and dilutions used for Western blotting.

Probe	Product information	Antibody type	Stock concentration	Dilution used	Band size (kDa)
INSR	Cell Signaling Technologies #23413 (RRID: AB_2924796)	Rabbit monoclonal	39 μg/ml	1:1000	95
GLO1	Abcam #ab137098 (RRID: AB_2818989)	Rabbit monoclonal	97 μg/ml	1:2000	21
AKT	Cell Signaling Technologies #9272 (RRID: AB_329827)	Rabbit polyclonal	31 μg/ml	1:1000	60
pAKT (Ser473)	Cell Signaling Technologies #9271 (RRID: AB_329825)	Rabbit polyclonal	10 μg/ml	1:1000	60
GAPDH	Cell Signaling Technologies #2118 (RRID AB_561053)	Rabbit monoclonal	42 μg/ml	1:4000	37

### 2.18 Quantitative RT-PCR

5-10 mg tissue samples were homogenized in 1 ml TRIzol (Invitrogen #15596026). RNA was isolated via chloroform-phenol extraction according to the manufacturer instructions. Quantitative RT-PCR was performed on RNA samples using the qScript XLT ToughMix system (QuantaBio #95132) with TaqMan gene expression FAM probes: IL6 (Mm01210732_g1); GAPDH (Mm99999915_g1). IL6 expression was calculated as fold change relative to GAPDH using the 2^∧^(-ΔΔC_T_) quantitation method (Supplemental File).

### 2.19 Statistical analysis

For mouse experiments, a sample size of 10 mice per group was determined by a power calculation for 80% power and significance of α = 0.05, based on variance from a preliminary glucose tolerance testing experiment. Ovaries from mice within these groups were divided into granulosa cell steroidogenesis and ovarian morphology analysis. Data were visualized and analyzed using GraphPad Prism for Windows, Version 11.0.0. Data are visualized on graphs as mean ± standard error. Each data point represents a biological replicate. Data normality was determined by the Shapiro-Wilk test. Normally distributed data were analyzed with parametric statistical tests (ordinary one-, two-, or three-way ANOVA, mixed-effects analysis); otherwise, non-parametric tests (Mann-Whitney, Kruskal-Wallis) were used as indicated in the figure captions. A significance of α = 0.05 was used for all tests. *Post-hoc* testing for multiple comparisons was performed for data sets with more than one variable as indicated in the figure captions.

## 3 Results

### 3.1 Developmental exposure to autoclaved rodent chow decreases body weight

**Figure 1:**
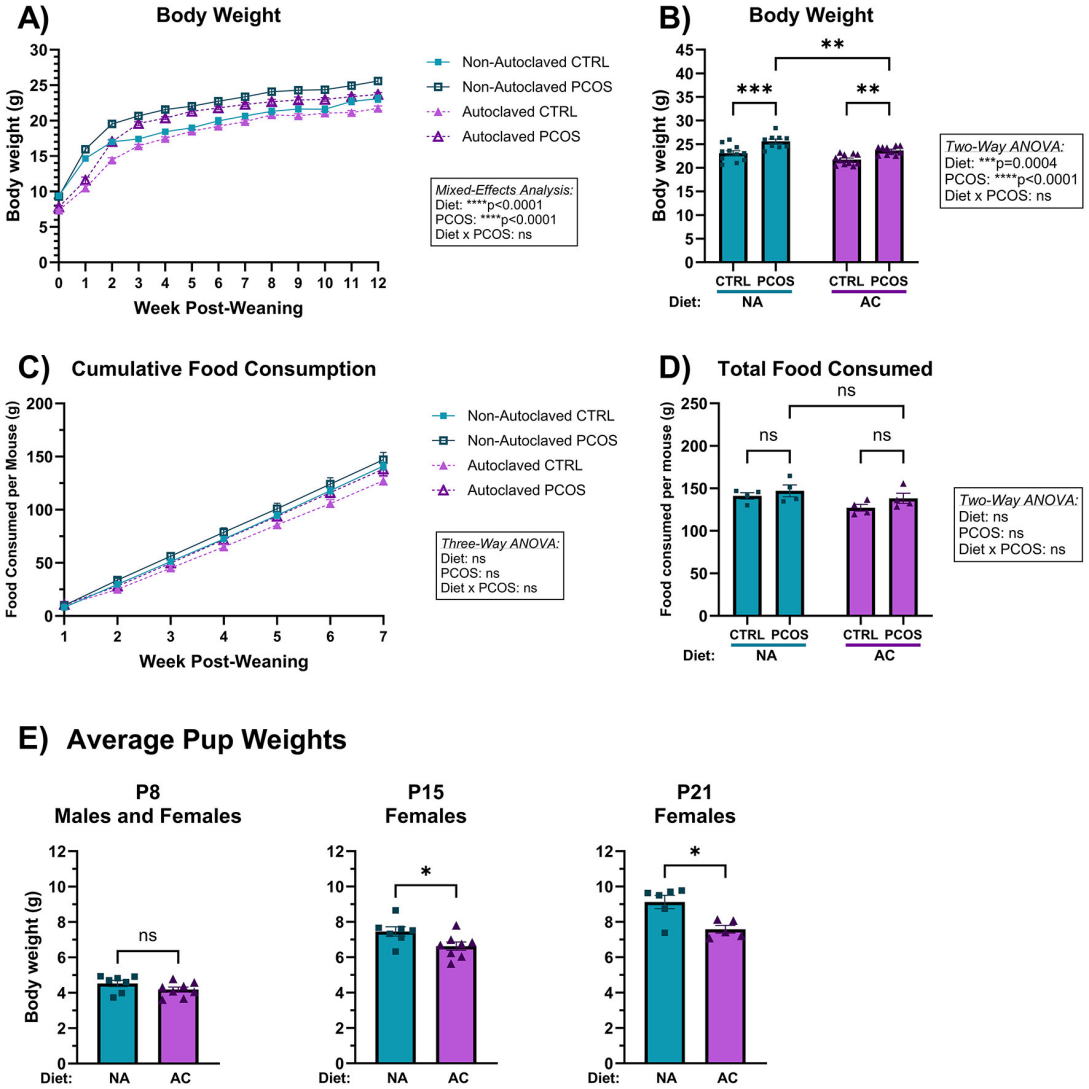
Effects of PCOS and developmental exposure to autoclaved chow on body weights and food consumption. PCOS was induced via chronic postnatal androgenization beginning at P21. All data shown represent female offspring only except where otherwise specified. **(A)** Body weight over time starting at weaning (P21). Results of mixed-effects analysis are shown on graph. **(B)** Body weight quantified at 15 weeks of age (12 weeks post-weaning). **(A, B)**: *n* = 9–11 mice per group. **(C)** Cumulative food consumption measured for 7 weeks starting at weaning. Results of three-way ANOVA are shown on graph. **(D)** Cumulative food consumption quantified at 7 weeks post-weaning. **(C, D)**: *n* = 4 cages per group. **(B, D)**: Ordinary two-way ANOVA results with uncorrected Fisher's LSD *post-hoc* comparisons are shown on graphs (α = 0.05). **(E)** Average pup weights by diet at P8, P15, and P21. *n* = 5–8 litters per diet; two-tailed Mann-Whitney test results shown on graphs. ns: not significant; *, *p* < 0.05; **, *p* < 0.01; ***, *p* < 0.001; ****, *p* < 0.0001. NA: Non-autoclaved; AC: Autoclaved.

Weight trajectories of PCOS and control mice fed autoclaved (AC) and non-autoclaved (NA) diets from pre-gestation through adulthood are shown in Figure 1A. PCOS mice in both diet groups gained significantly more weight than control mice, but the weight difference was smaller on the autoclaved chow. At 12 weeks post-weaning (15 weeks of age), body weights of PCOS mice on AC chow were significantly lower than PCOS mice on NA chow (Figure 1B). Food consumption was measured as cumulative food intake beginning at weaning (P21) through 7 weeks post-weaning. Cumulative food consumption was approximately 8% lower in both control and PCOS mice on AC chow compared with NA chow, but this effect was not statistically significant. Food intake was not significantly different in PCOS mice compared to controls on either diet (Figures 1C, D). Litter averages of pup body weights were measured as well: pups born to dams on AC chow had similar weights to NA chow pups on P8 (both sexes combined due to young age of pups), however on both P15 and P21 pups were significantly smaller when dams were exposed to the AC chow compared to NA chow (female only pups) (Figure 1E). Overall, these data show that autoclaved chow caused a decrease in juvenile and adult body weight, attenuating the PCOS-induced weight gain in adulthood, despite similar food consumption.

### 3.2 Autoclaved chow improves adiposity, hyperinsulinemia, and glucose tolerance in insulin-resistant PCOS mice

**Figure 2:**
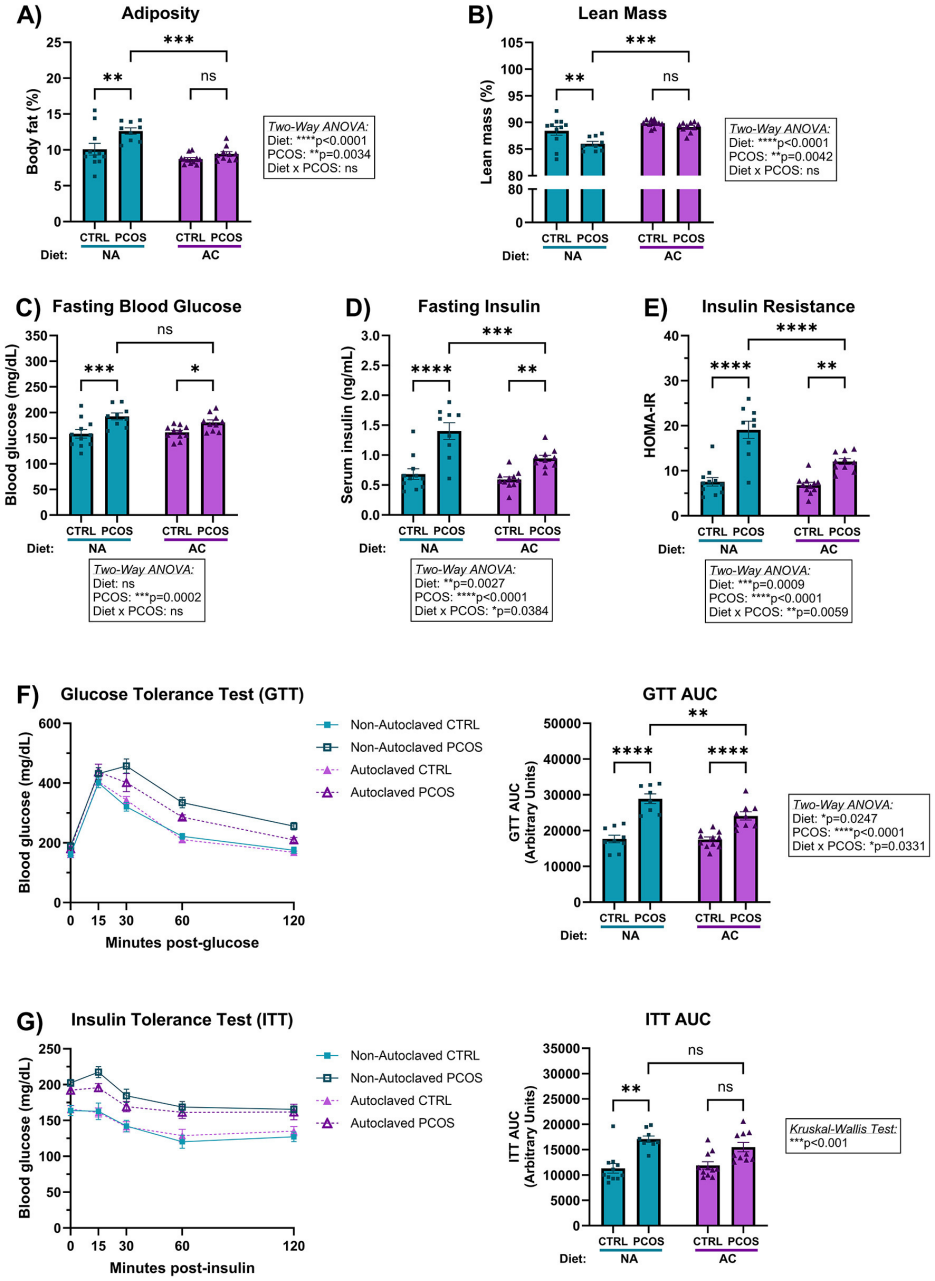
Metabolic parameters of experimental mice. **(A)** Body fat percentage (adiposity) and **(B)** lean mass percentage, both measured by DEXA at 14–15 weeks of age. **(C)** 6-h fasting blood glucose as measured during glucose tolerance test (GTT) at 14–15 weeks of age. **(D)** 6-h fasting insulin as measured via ELISA using serum collected at sacrifice (16 weeks of age). **(E)** Insulin resistance quantified as HOMA-IR from fasting glucose **(C)** and insulin **(D)** values. **(F)** Glucose clearance as measured by intraperitoneal GTT (2 g/kg i.*p*. glucose following a 6-h fast) at 14–15 weeks of age. GTT quantified as area under the curve (AUC); baseline = 100 mg/dl. **(G)** Insulin-stimulated glucose clearance as measured by intraperitoneal insulin tolerance test (ITT) (0.5 U/kg i.*p*. insulin following a 6-h fast) at 14–15 weeks of age. ITT quantified as AUC; baseline = 40 mg/dl. *n* = 8–11 mice per group for all panels. Where indicated, data were analyzed by ordinary two-way ANOVA with uncorrected Fisher's LSD *post-hoc* test for multiple comparisons. ns: not significant; *, *p* < 0.05; **, *p* < 0.01; ***, *p* < 0.001; ****, *p* < 0.0001. NA: Non-autoclaved; AC: Autoclaved; DEXA: Dual Energy X-ray Absorptiometry; HOMA-IR: Homeostatic Model Assessment of Insulin Resistance.

Body composition (fat and lean mass) was measured with dual energy X-ray absorptiometry (DEXA) and shown in Figures 2A, B. PCOS mice had increased adiposity (fat percent) and reduced lean body mass (lean percent) compared to control mice in the NA diet group. However, on the AC diet, PCOS mice had similar fat mass and lean mass as control mice. Increased adiposity is associated with systemic insulin resistance, therefore we next measured glucose metabolism. PCOS mice exhibited elevated fasting blood glucose compared to controls in both diet groups (Figure 2C). PCOS mice also had significantly higher fasting serum insulin than controls in the NA chow group, while in the AC chow group, PCOS mice had a smaller increase in fasting insulin over control mice, and PCOS mice on AC chow had lower fasting insulin than PCOS mice on NA chow (Figure 2D). Insulin resistance, calculated from fasting glucose and insulin values as homeostatic model assessment of insulin resistance (HOMA-IR), was higher in PCOS mice compared to controls on both diets, but this difference was smaller in mice fed AC chow than NA chow, and PCOS mice on AC chow had lower HOMA-IR index than PCOS mice on NA chow (Figure 2E). Glucose tolerance testing (GTT), quantified as area under the curve (AUC), showed significantly impaired glucose tolerance in PCOS mice compared with control mice on both diets, however PCOS mice on the AC diet had lower AUC than PCOS mice on the NA diet (Figure 2F). Insulin tolerance testing (ITT) was quantified similarly to GTT. Insulin-stimulated glucose clearance was decreased in PCOS mice compared with controls on the NA diet, indicative of systemic insulin resistance. On the AC diet, however, PCOS mice did not show a significant increase in insulin-stimulated glucose clearance compared to control mice. No difference in AUC after insulin injection was observed between PCOS mice on AC diet and PCOS mice on NA diet (Figure 2G). In summary, control mice fed the AC diet had similar metabolic characteristics as control mice fed the NA diet, however in PCOS mice the AC diet led to reduced adiposity, higher lean body mass and improved systemic insulin sensitivity compared to the NA diet.

### 3.3 Autoclaved chow does not affect the reproductive manifestations of PCOS mice

**Figure 3:**
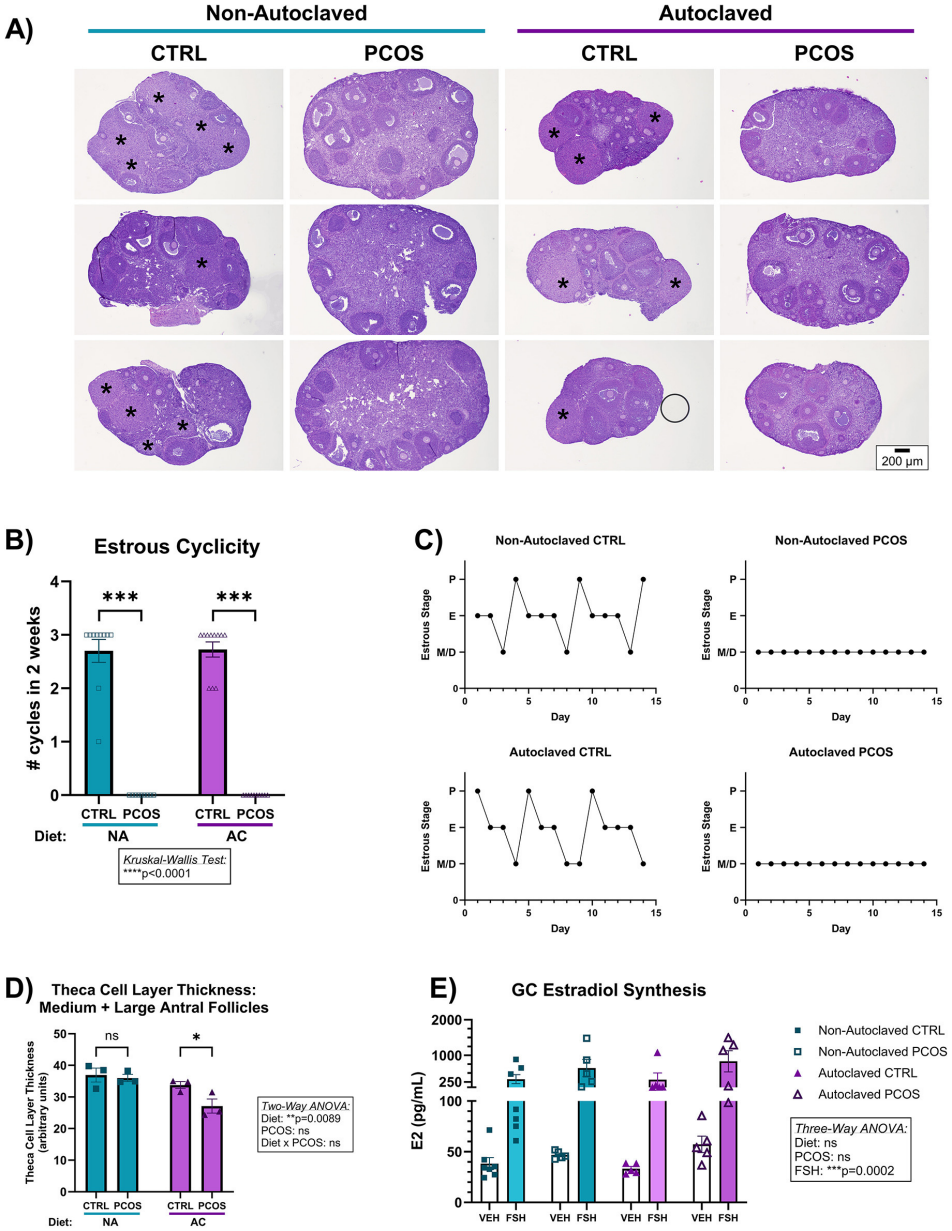
Reproductive characterization of the postnatal androgenization PCOS mouse model on autoclaved and non-autoclaved chow diets. **(A)** Hematoxylin- and eosin-stained sections of mouse ovaries from 3 mice per group, dissected at 16 weeks of age. Scale bar = 200 μm; corpora lutea denoted by black asterisks. **(B)** Quantification of estrous cyclicity as measured in mice for 14 days via daily vaginal lavage. An estrous cycle is defined as P followed directly by E. *n* = 9–11 mice per group. Analysis by Kruskal-Wallis test and Dunn's multiple comparisons is shown. **(C)** Representative estrous cycles of mice from each group. P: proestrus, E: estrus, M/D: metestrus/diestrus. **(D)** Theca cell layer thickness of medium and large antral follicles in mouse ovarian sections. Graphed data points represent averages of all non-atretic antral follicles in one random section for each mouse. *n* = 3 mice per group. Analysis by two-way ANOVA (α = 0.05) and uncorrected Fisher's LSD multiple comparisons is shown. **(E)** Granulosa cell (GC) steroidogenesis was assayed by FSH-stimulated estradiol (E2) secretion of cultured primary mouse GCs. All samples, including vehicle wells, were supplied with 50 nM testosterone as E2 substrate. *n* = 5–7 mice per group. Results shown for three-way ANOVA (α = 0.05) matched by mouse; interaction effects (not shown) all ns. ns: not significant; *, *p* < 0.05; **, *p* < 0.01; ***, *p* < 0.001; ****, *p* < 0.0001. NA: Non-autoclaved; AC: Autoclaved.

Figure 3 shows the reproductive parameters of experimental mice. PCOS mice in both diet groups had no corpora lutea observed in ovarian sections (Figure 3A) and developed persistent anestrus with zero estrous cycles observed over 14 days (Figures 3B, C), indicating anovulation. Theca cell layer thickness in antral follicles of PCOS mice was similar to controls on the NA diet, but slightly reduced in PCOS mice compared to controls on the AC diet (Figure 3D). Primary granulosa cells were cultured and treated with FSH. Estradiol synthesis was significantly induced by FSH in both PCOS and control mice on both NA and AC diets, with no differences attributed to diet or PCOS (Figure 3E). Overall, these results suggest that the autoclaved diet improves the metabolic, but not the reproductive manifestations of PCOS in this mouse model.

### 3.4 Autoclaving sharply decreases sugar content and induces methylglyoxal in rodent chow without affecting macronutrient levels

**Figure 4:**
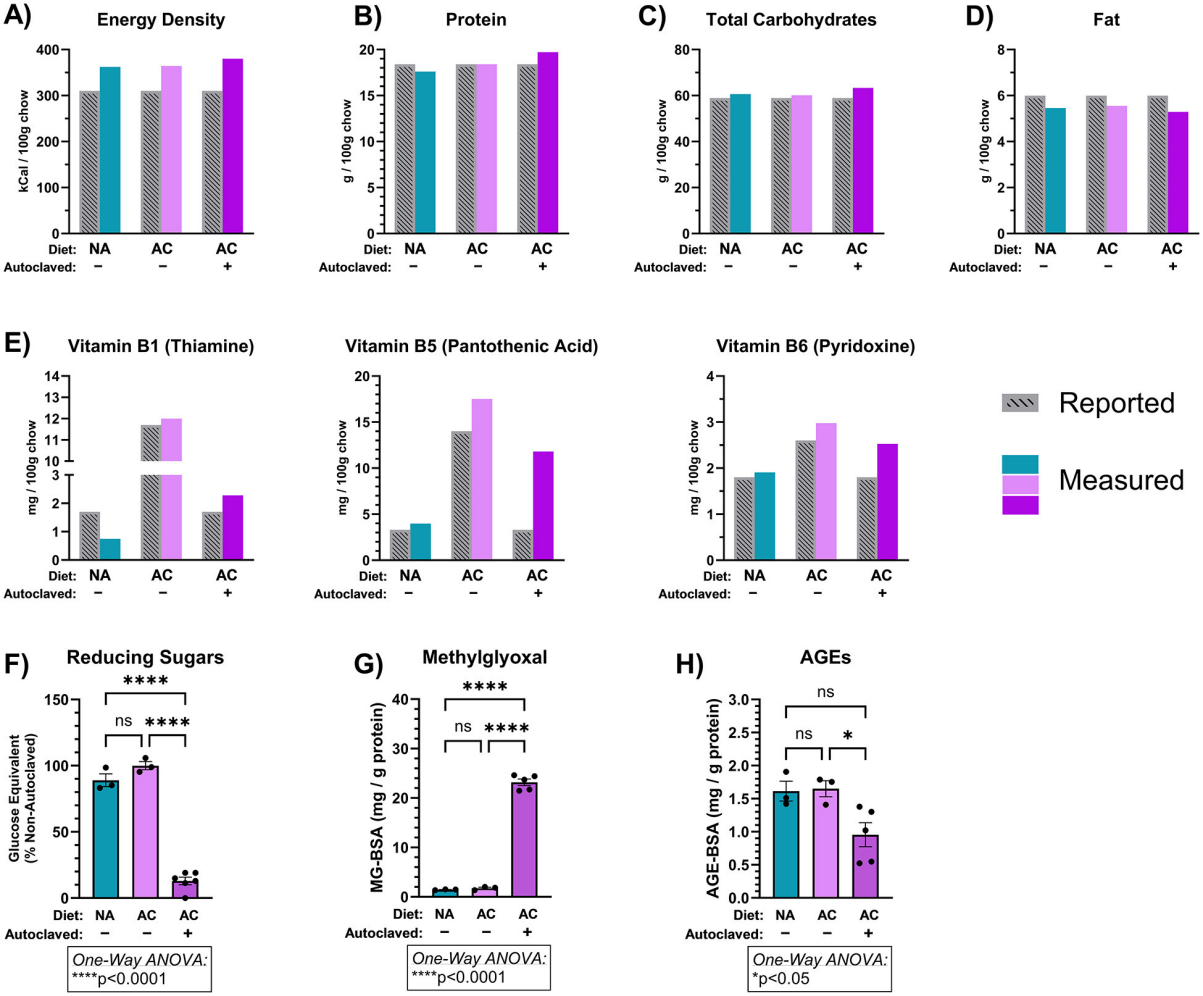
Characterization of select experimental diet parameters. **(A-D)** Energy density (caloric content) and macronutrient (carbohydrate, protein, and fat) content, along with select B vitamins **(E)**, were quantified in chow samples by an independent food testing laboratory (solid-colored bars) and compared to manufacturer-reported levels (gray patterned bars). *n* = 1 reading per group. **(F)** Reducing sugars were measured in chow extracts using a quantitative adaptation of Benedict's test. **(G)** Methylglyoxal and **(H)** AGEs were quantified in chow extracts via ELISA. **(F-H)**: *n* = 3-5 chow pellets per group; ordinary one-way ANOVA results shown with Tukey's multiple comparisons test. ns: not significant; *, *p* < 0.05; ***, *p* < 0.001; ****, *p* < 0.0001. NA: Non-autoclaved; AC: Autoclaved.

To understand the unexpected effects of autoclaved chow on body weight and metabolic parameters in PCOS mice, we next characterized the effects of autoclaving on rodent diet nutrient and toxicant composition. Samples from each diet were analyzed for energy density (caloric content), carbohydrate, protein, and fat by a commercial food testing laboratory. The measured macronutrient levels were comparable to manufacturer-reported levels in both the AC and NA diets. Additionally, measured macronutrients were similar between diets and were not affected by autoclaving (Figures 4A–D).

The AC chow was supplemented with higher levels of certain vitamins to protect against autoclave heat degradation, however we found that AC diet caused juvenile pup growth restriction compared to NA diet (Figure 1E). Therefore, to rule out vitamin deficiency, we measured chow levels of heat-sensitive vitamins B1, B5, and B6, which play critical roles in growth and metabolism (52). Although multiple discrepancies between reported and measured B vitamin levels were identified, the measured levels of these B vitamins were sufficient according to rodent dietary requirements determined by the National Research Council (53), and in fact the AC chow had higher levels of all three vitamins than the NA chow (Figure 4E).

We next measured reducing sugar content in the research diets. Autoclaving vastly depleted reducing sugar content in chow extract, resulting in very low sugar content in AC chow compared with NA chow (Figure 4F). To evaluate heat-induced toxicants, we measured advanced glycation end products (AGEs) and methylglyoxal (MG). MG increased dramatically in the AC chow and was significantly greater in AC chow than in NA chow, while AGE content was slightly decreased in AC chow after autoclaving (Figures 4G, H). These results suggest that autoclaving converts a significant amount of reducing sugars to MG in this diet without impacting macronutrient content or generating additional AGEs.

### 3.5 Autoclaved chow ablates PCOS effects on liver insulin receptor and glyoxalase 1 expression

**Figure 5:**
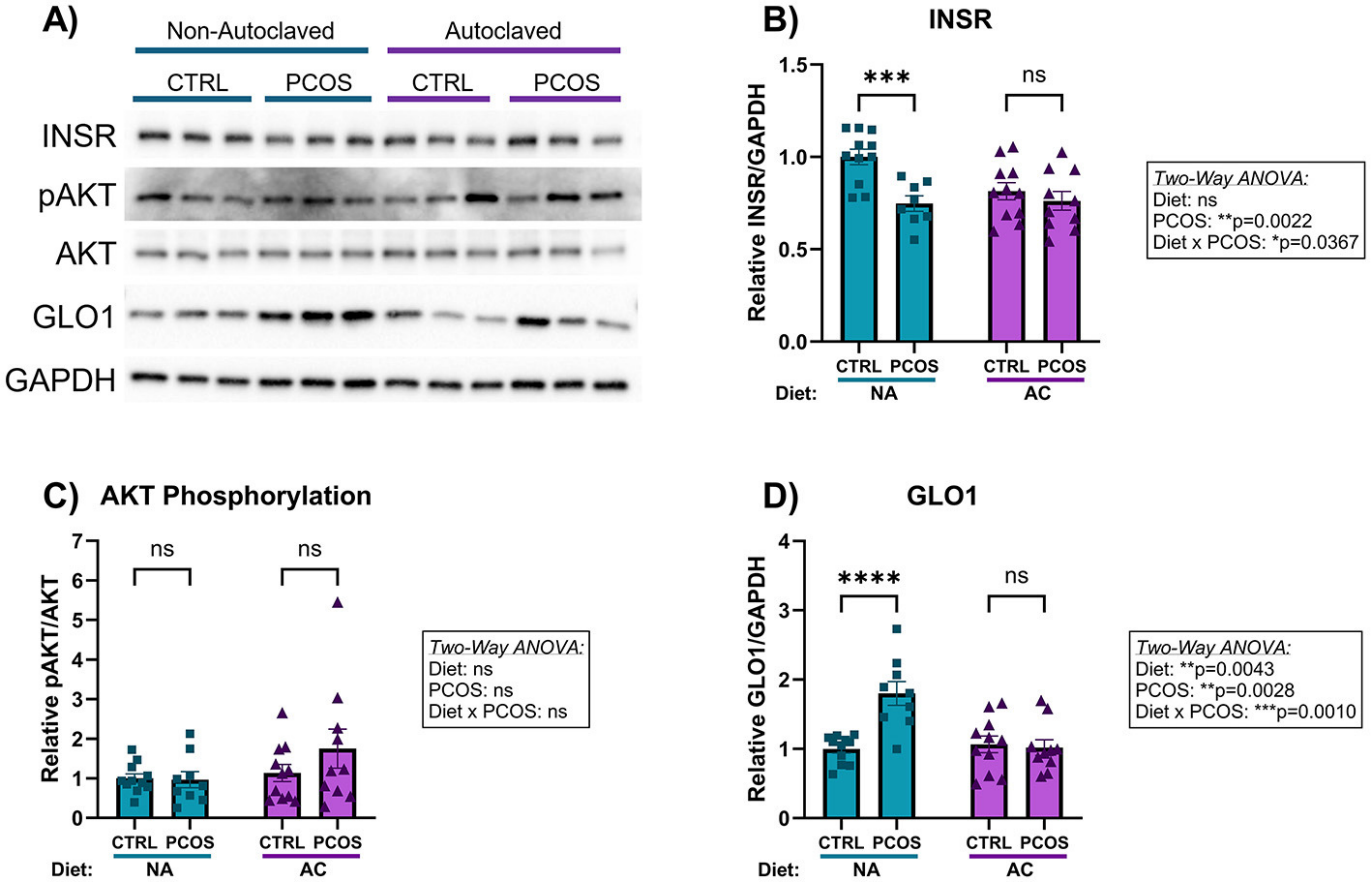
Western blot analysis of protein expression in whole liver from 6-h fasted experimental mice. **(A)** Representative Western blot images of liver samples. **(B)** Relative band density of INSR. **(C)** Relative AKT phosphorylation. **(D)** Relative band density of GLO1. *n* = 9-11 mice per group. Ordinary two-way ANOVA (α = 0.05) results shown with uncorrected Fisher's LSD *post-hoc* test for multiple comparisons. ns: not significant; *, *p* < 0.05; **, *p* < 0.01; ***, *p* < 0.001; ****, *p* < 0.0001. NA: Non-autoclaved; AC: Autoclaved.

Chow characterization experiments showed a decrease in dietary sugar content due to autoclaving, which may affect insulin signaling and glucose metabolism. Therefore, we next used Western blotting to measure fasted protein expression of insulin receptor (INSR) and phosphorylation of downstream kinase AKT (protein kinase B) in metabolically active tissues (liver, skeletal muscle, and adipose). Liver INSR expression was decreased in PCOS mice on NA chow, but on AC, liver INSR was similar in PCOS and control mice (Figures 5A, B). Liver AKT phosphorylation was not affected by diet or PCOS (Figure 5C). To investigate the effects of high MG in the AC chow (Figure 4G), we measured the detoxifying enzyme GLO1. Liver GLO1 expression was increased in PCOS mice on NA chow, but on AC chow, liver GLO1 was similar in PCOS and control mice (Figure 5D). In summary, whole liver protein analysis showed a PCOS-induced decrease in INSR and increase in GLO1 on NA chow, while PCOS and control mice had similar liver protein expression on the AC chow.

### 3.6 Autoclaved chow reduces skeletal muscle INSR expression and AKT phosphorylation in PCOS

**Figure 6:**
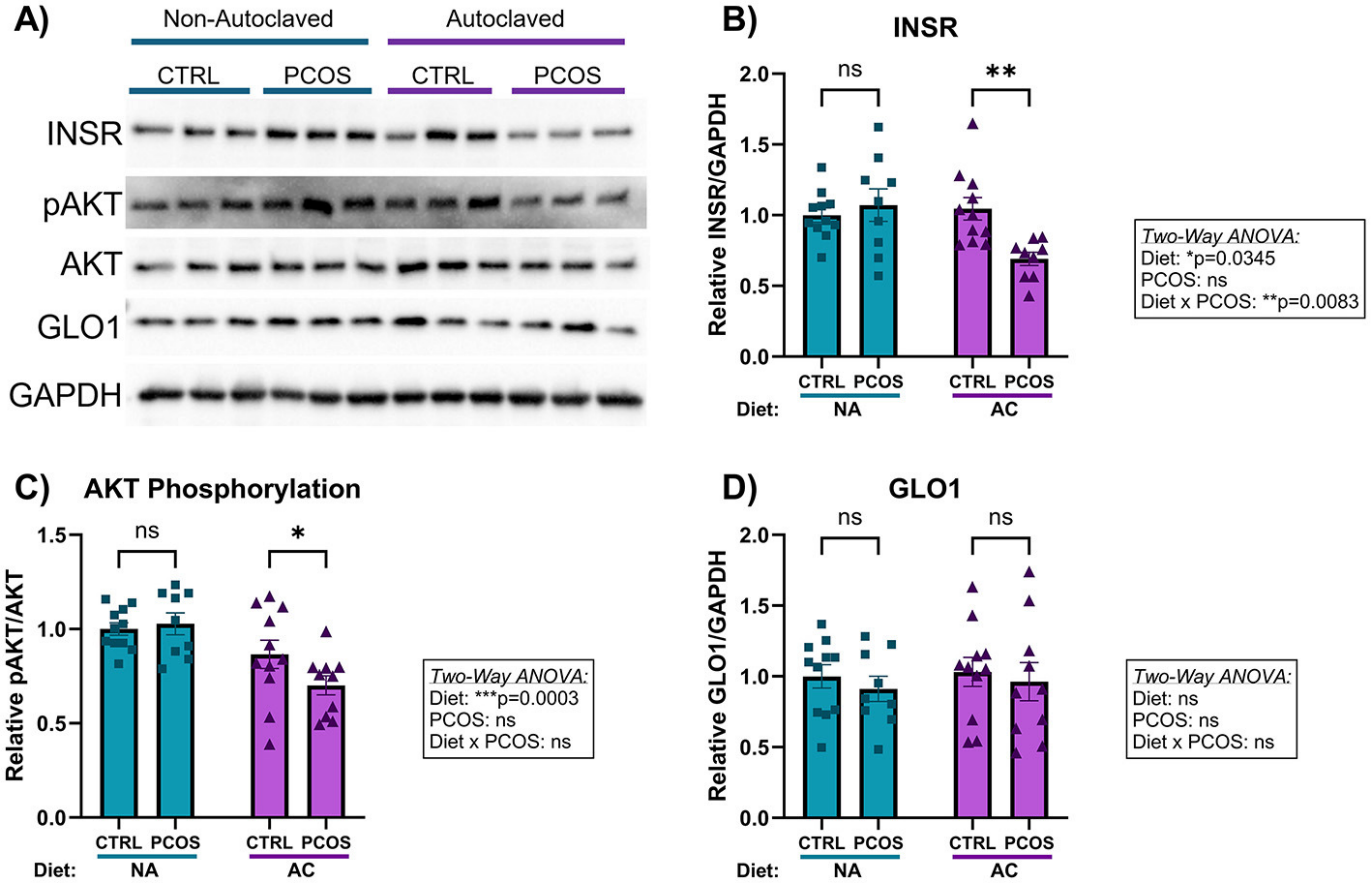
Western blot analysis of protein expression in quadriceps muscle from 6-h fasted experimental mice. **(A)** Representative Western blot images of skeletal muscle samples. **(B)** Relative band density of INSR. **(C)** Relative AKT phosphorylation. **(D)** Relative band density of GLO1. *n* = 9–11 mice per group. Ordinary two-way ANOVA (α = 0.05) results shown with uncorrected Fisher's LSD *post-hoc* test for multiple comparisons. ns: not significant; *, *p* < 0.05; **, *p* < 0.01; ***, *p* < 0.001; ****, *p* < 0.0001. NA: Non-autoclaved; AC: Autoclaved.

Skeletal muscle is an important metabolic tissue due to its high energy demand and large role in glucose uptake. In the NA chow group, muscle INSR expression was unchanged in PCOS mice compared with controls, but in the AC chow group, INSR was decreased in PCOS mice compared with controls (Figures 6A, B). Similarly, muscle AKT phosphorylation was similar in PCOS mice compared with controls fed NA chow and reduced in PCOS mice compared with controls fed AC chow (Figure 6C). No diet or PCOS effects were found in GLO1 expression in muscle tissue (Figure 6D).

### 3.7 Autoclaved chow attenuates PCOS-induced elevation of AKT phosphorylation in adipose tissue

**Figure 7:**
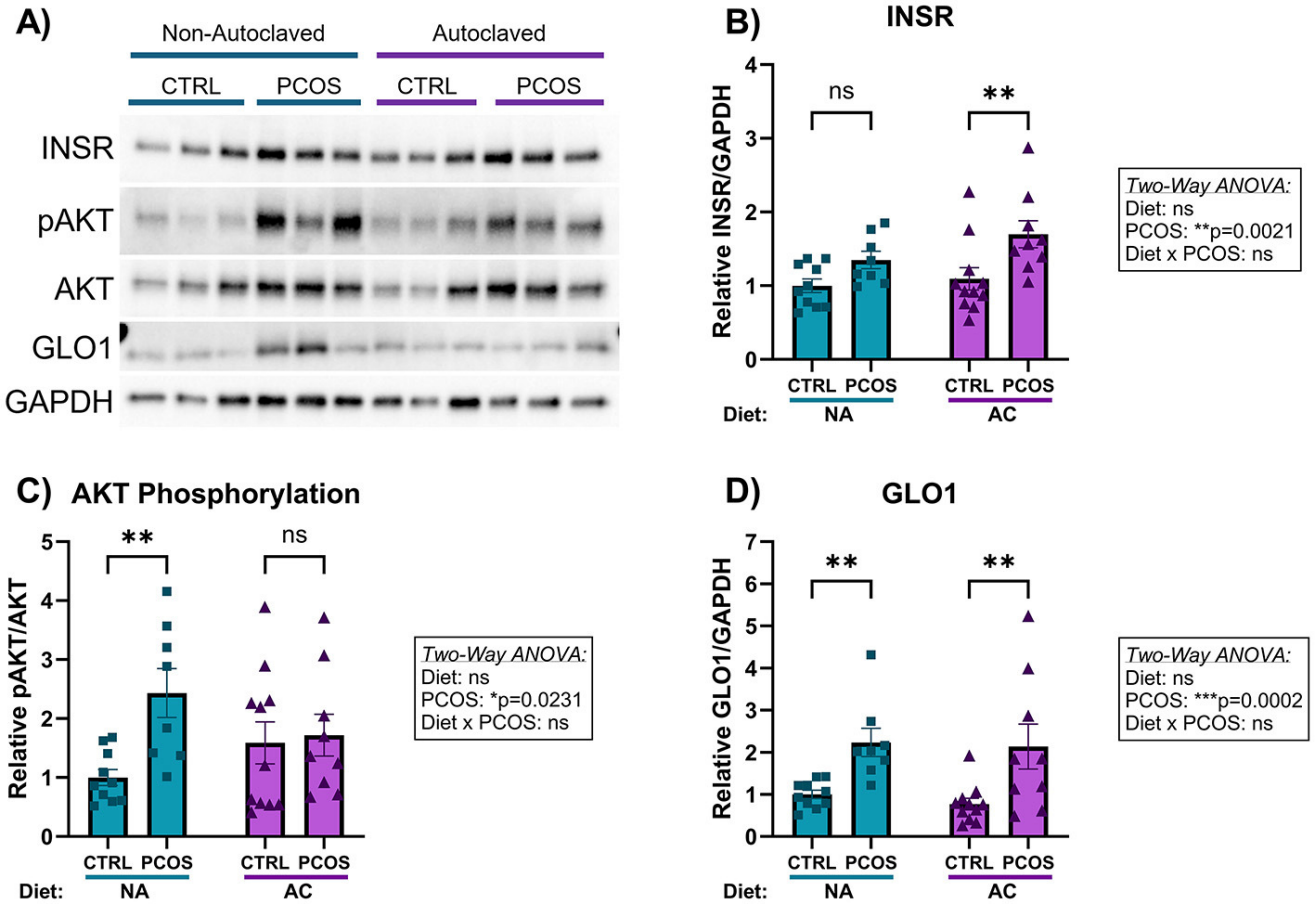
Western blot analysis of protein expression in perirenal adipose from 6-h fasted experimental mice. **(A)** Representative Western blot images of adipose samples. **(B)** Relative band density of INSR. **(C)** Relative AKT phosphorylation. **(D)** Relative band density of GLO1. *n* = 8–11 mice per group. Ordinary two-way ANOVA (α = 0.05) results shown with uncorrected Fisher's LSD *post-hoc* test for multiple comparisons. ns: not significant; *, *p* < 0.05; **, *p* < 0.01; ***, *p* < 0.001; ****, *p* < 0.0001. NA: Non-autoclaved; AC: Autoclaved.

Adipose tissue is involved in numerous regulatory processes concerning both metabolism and inflammation. Therefore we assayed metabolic markers and GLO1 expression in perirenal adipose tissue from fasted experimental mice. In the NA chow group, adipose INSR expression was similar in control and PCOS mice; however, in the AC chow group, adipose INSR was significantly higher in PCOS mice compared to controls (Figures 7A, B). Fasting AKT phosphorylation was increased with PCOS mice compared with controls on NA chow, but it was similar in PCOS and control mice on AC chow (Figure 7C). We also found a significant increase in adipose GLO1 expression in PCOS mice compared with control mice on both diets (Figure 7D). These results suggest that adipose GLO1 may be upregulated in response to metabolic dysregulation, and that diet alters the white adipose tissue response to androgen exposure in our PCOS mouse model.

### 3.8 PCOS induces adipose IL-6 mRNA transcription, which is improved by autoclaved chow

**Figure 8:**
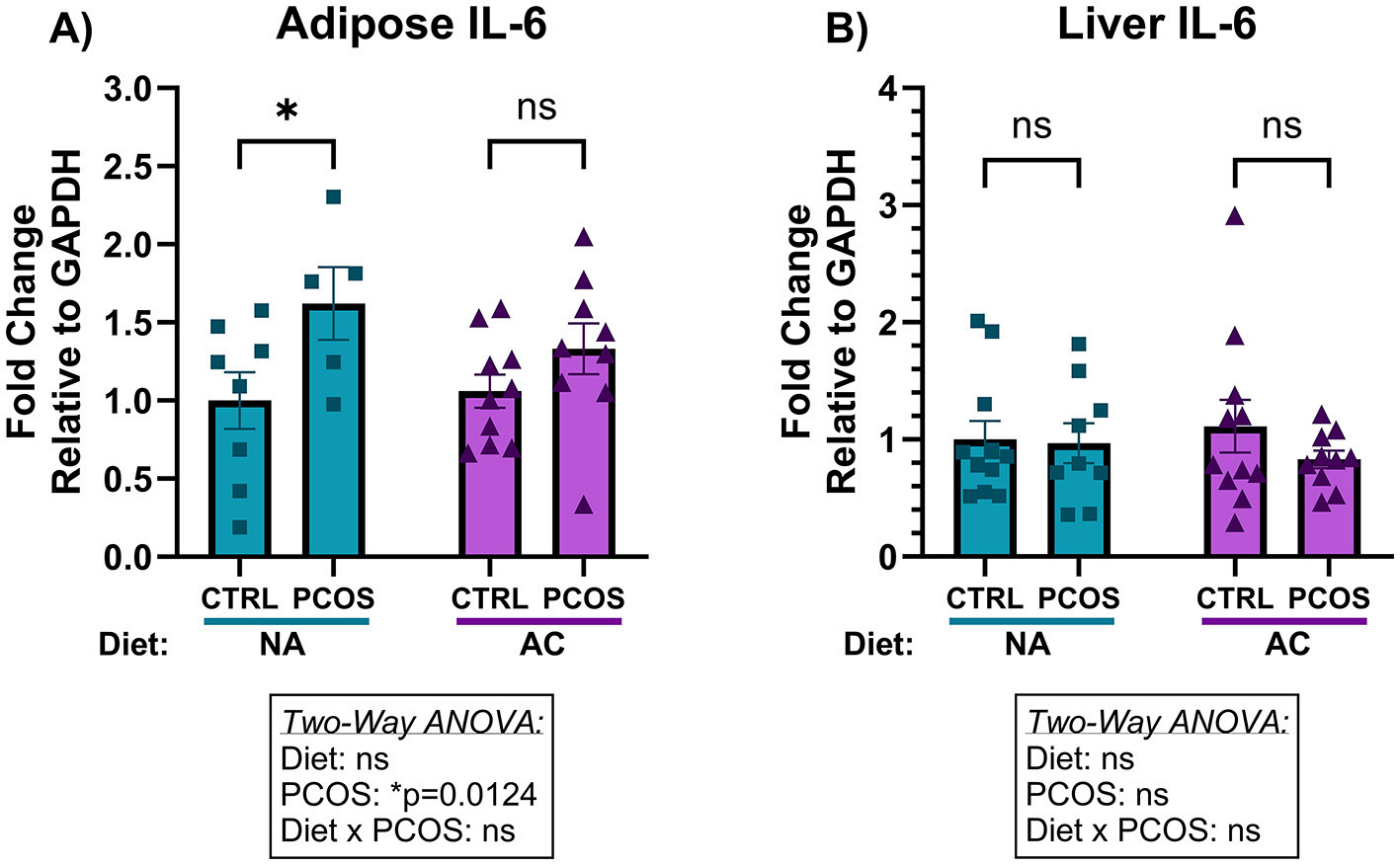
qPCR quantification of IL-6 mRNA transcription in whole tissues isolated from 6-h fasted mice. **(A)** Perirenal adipose IL-6 mRNA expression, *n* = 5–11 mice per group. **(B)** liver IL-6 mRNA expression, *n* = 9–11 mice per group. Ordinary two-way ANOVA (α = 0.05) results shown with uncorrected Fisher's LSD *post-hoc* test for multiple comparisons. ns: not significant; *, *p* < 0.05. NA: Non-autoclaved; AC: Autoclaved.

To examine tissue-level inflammation, we measured IL-6 mRNA expression by qPCR in adipose tissue and liver of experimental mice. We observed an increase in IL-6 transcription in PCOS mouse perirenal adipose tissue compared with control mice in the NA diet group, while in the AC diet group, adipose IL-6 expression was similar in control and PCOS mice (Figure 8A). PCOS did not affect liver IL-6 expression in either diet group (Figure 8B). These data suggest that macrophage-mediated adipose tissue inflammation may be improved by AC diet in our PCOS mouse model, while this process may not play an important role in the liver.

## 4 Discussion

We found that autoclaving a rodent diet, a common and widespread method in research animal husbandry, significantly improved the metabolic outcomes of a well-characterized mouse model of polycystic ovary syndrome (PCOS). Consistent with previous literature (44, 45), DHT-treated PCOS mice developed increased adiposity and systemic insulin resistance despite consuming a similar amount of food as controls on both experimental diets. In this report, we show that autoclave processing of a commercially available, autoclavable rodent diet significantly reduced its sugar content. Further, exposure to this autoclaved (AC) chow unexpectedly reduced excess adiposity and improved glucose metabolism in PCOS mice. Briefly, metabolic characterization demonstrated improvements in hyperinsulinemia, glucose tolerance, and insulin-stimulated glucose uptake in PCOS mice that were fed the AC chow, but no diet-induced improvement in fasting glucose was observed. These results suggest that the AC chow improves both postprandial insulin secretion and systemic insulin sensitivity. Analysis of mouse tissue revealed that autoclaving reduces the PCOS-related increases in liver glyoxalase 1 (GLO1) expression, skeletal muscle AKT phosphorylation and insulin receptor (INSR) expression, and adipose IL-6 expression.

Mice consumed approximately 8% less of the AC chow compared to the non-autoclaved (NA) chow. We noted that autoclaving increased chow hardness, which has been shown to decrease food consumption and developmental growth in rodents (54). The AC diet led to reduced body weights in P15 and P21 pups, which could be caused by this small reduction in food intake by pregnant dams. However, the developmental growth restriction that we observed on the AC chow is overcome by early adulthood (approximately 6 weeks of age), and no other developmental or behavioral derangements were observed in offspring exposed to either diet.

The described diets were selected for this study because they were designed by the manufacturer to be nutrient-matched after autoclaving. However, we were unable to obtain exact autoclaving recommendations for the supplemented chow, prompting characterization of the autoclave effects on diet composition. As stated by the research diet manufacturer, the AC and NA diets had equivalent macronutrient content. However, we found significant differences in reducing sugars between the autoclaved and nutrient-matched non-autoclaved diets, as well as discrepancies between the manufacturer-reported and measured levels of vitamins B1, B5, and B6. These findings highlight the importance of independent validation of manufacturer claims regarding nutritive content. While excess B vitamins are unlikely to be of consequence to insulin signaling, depletion of sugar content is the most likely factor behind the observed metabolic differences in PCOS mice on the NA and AC diets in this study. Our findings suggest that chow pasteurization methods may be differentially impacting research outcomes.

Autoclaving was found to significantly increase methylglyoxal (MG) in the chow, suggesting that heat degradation of sugar in the AC diet resulted in the production of MG, a reactive dicarbonyl with cytotoxic properties (55). MG causes oxidative stress in tissues and pathologically adducts to biomolecules, ultimately forming advanced glycation end products (AGEs). Dicarbonyls and AGEs, as well as the cellular stress responses they activate, are associated with adverse effects on metabolism and many other pathologies (56, 57). We did not detect AGE induction with autoclaving; in fact, AGEs unexpectedly decreased in the AC chow. This may be due to relatively high levels of AGE inhibitors such as vitamin B6, which we measured to be over 10 times the minimal recommended amount for murine maintenance and growth in the autoclaved chow, or other unknown factors (53, 58). In this experiment, it appears that any metabolic disruption caused by MG toxicity may be outweighed or confounded by another heat-induced effect: the degradation of simple carbohydrates. The metabolic improvement in PCOS-induced insulin resistance that we observed from the AC chow may therefore be an effect of a low-glycemic index, macronutrient-matched diet. It is well-known that a low-glycemic diet improves insulin sensitivity, glucose tolerance, and hyperinsulinemia (59), consistent with the results presented here. The metabolic benefits of reducing dietary sugar intake may also be potentiated by the slight but non-significant decrease in food consumption on the AC chow.

To better understand how heat-processed diet affects insulin signaling in PCOS mice, we evaluated protein expression of insulin signaling markers in metabolic tissues from these mice. PCOS induced tissue-specific changes in INSR expression and AKT phosphorylation. On the NA diet, PCOS induced liver INSR expression patterns consistent with hepatic insulin resistance, as previously reported in this PCOS mouse model (45). Despite this, AKT phosphorylation was similar in PCOS and control mice on NA diet, suggesting that hyperinsulinemia or other growth factor signaling in these PCOS mice overcomes hepatic insulin resistance. By contrast, the AC diet ablated these PCOS-induced patterns in liver, reflecting an attenuation of systemic insulin resistance in PCOS mice on this diet compared with the NA diet. In the skeletal muscle, PCOS did not alter INSR and phosphorylated AKT on the NA diet, indicating that muscle insulin resistance in these PCOS mice may be due to post-receptor signaling defects, but compensated by hyperinsulinemia. By contrast, both of these markers were reduced in PCOS mice compared with controls on the AC diet, suggesting that PCOS mice on this diet develop some degree of skeletal muscle insulin resistance, but this is not reflected by their systemic glucose metabolism, perhaps due to increased glucose uptake by other tissues. In the adipose tissue, PCOS mice on the NA diet showed similar INSR levels but higher AKT phosphorylation than controls, suggesting that hyperinsulinemia in these PCOS mice results in increased adipocyte metabolic activity. However, in mice on the AC diet, PCOS had a different effect on adipocytes, where INSR expression was increased, but AKT phosphorylation was similar to controls, suggesting that adipocyte metabolism, along with systemic glucose metabolism and circulating insulin level, may not be as dramatically altered by PCOS on this diet as it is on the NA diet. Notably, INSR retains a robust receptor reserve, with 90% of total receptors considered “spare receptors,” resulting in the potential for adequate signaling responses even with low levels of receptor expression (60, 61). As a result, small effect sizes in INSR expression such as those observed here may not be responsible for the observed diet impact on systemic metabolism. One of the limitations of this study is that ovarian insulin signaling was not examined.

In our study, simple sugars were likely converted into MG during autoclaving of the mouse food. MG is detoxified by GLO1, which is initially upregulated by dicarbonyl stress, but is downregulated or inactivated due to prolonged dicarbonyl exposure and depletion of the GLO1 cofactor glutathione in type 2 diabetes and PCOS (43, 62–64). In our experiments, liver GLO1 expression was increased in PCOS mice compared to controls on the NA diet, suggesting that the liver plays an important role in detoxifying PCOS-related endogenous dicarbonyl formation. However, on the AC diet, there was no upregulation of GLO1 in the livers of PCOS mice compared to controls, possibly due to high dietary MG overwhelming the liver glyoxalase system's reductive capacity (65). In the adipose tissue, GLO1 was upregulated in PCOS mice compared with controls on both the NA and AC diets, demonstrating dicarbonyl adipose toxicity due to chronic hyperandrogenism, and increased GLO1 expression which was not affected by the high-MG diet. These findings suggest that tissue differences in response to chronic dicarbonyl exposure may lead to tissue-specific adaptations, where liver GLO1 upregulation may require additional cofactors, such as glutathione. One of the limitations of this study is that GLO1 activity was not evaluated, and may not be accurately represented by its expression levels.

Dicarbonyl stress and oxidative stress often co-occur, and both are considered to be major drivers of metabolic dysfunction in PCOS (43, 66–68). We consequently examined inflammation via IL-6 mRNA expression in the liver and adipose tissues, where we observed PCOS-induced increases in GLO1 expression. On the NA diet, IL-6 mRNA expression was increased in the adipose tissue of PCOS mice compared with controls, indicating that adipose resident macrophage activation accompanies increased adipocyte metabolic activity and excess adiposity in this model of PCOS and may be one of the underlying disease mechanisms leading to insulin resistance. On the AC diet, however, both adipose AKT phosphorylation and systemic insulin sensitivity were similar in PCOS and control mice. IL-6 expression was also not significantly increased in PCOS adipose compared with control mice on the AC diet. These findings suggest that dietary factors leading to differential PCOS metabolic manifestations in this study may be mediated by adipose tissue responses to dietary sugar content.

While this study did not fully elucidate the molecular pathways through which autoclaving food reduced the metabolic manifestations of PCOS, we found consistently that tissue markers of insulin signaling were differentially affected by PCOS on the AC compared to NA diet. This has broad implications on the widespread practice of research animal diet autoclave pasteurization. Consideration should be taken when choosing or changing methods of diet preparation. Manufacturers of chow diets are not required to accurately report changes in the sources of ingredients or batch-to-batch variation in their products (5, 69). Variation is therefore intrinsic to chow diets. This variation is little-known and frequently unreported, yet it is undoubtedly at least partially to blame for replicability and reproducibility issues in animal studies. Future experiments will focus on elucidating our unexplained findings and more thoroughly characterizing autoclave effects on diet composition as well as the systemic and tissue-level effects the autoclaved diet may be exerting on development, inflammation, and insulin signaling.

In conclusion, dietary modulation in animal studies presents numerous challenges and considerations. We found that an autoclaved chow diet unexpectedly improved metabolic outcomes in insulin-resistant PCOS mice, which may be due to the heat degradation of dietary sugars. We provide further evidence that autoclave sterilization of rodent chow, a widely-accepted practice in animal research, produces complex changes in diet composition that can significantly impact research outcomes. We recommend more robust consistency and reporting standards of research diets, urging investigators to exercise caution whenever dietary parameters are altered between, during, or as part of animal studies.

## Data Availability

The original contributions presented in the study are included in the article/Supplementary material; additional information will be provided upon request by the corresponding author without undue reservation.
